# Acute Bacterial Septicemia in a Critically Endangered Roloway Monkey in a Primate Breeding Center at the Accra Zoological Garden, Ghana: A Case Report

**DOI:** 10.1155/crve/4924170

**Published:** 2026-01-07

**Authors:** Richard Suu-Ire, Henry Abugri, Samuel Asumah, Richard Abbiw, David Turkson, Mustapha Ahmed, Peter Gathumbi

**Affiliations:** ^1^ School of Veterinary Medicine, University of Ghana, Accra, Ghana, ug.edu.gh; ^2^ Wildlife Division, Forestry Commission, Accra, Ghana; ^3^ West African Centre for Cell Biology of Infectious Pathogens, University of Ghana, Accra, Ghana, ug.edu.gh; ^4^ Department of Veterinary Pathology Microbiology and Parasitology, Faculty of Veterinary Medicine, University of Nairobi, Nairobi, Kenya, uonbi.ac.ke

**Keywords:** *β*-hemolytic *Streptococcus*, captive breeding, *Cercopithecus roloway*, endangered primate, Ghana, leiomyosarcoma, pneumonia, septicemia

## Abstract

This case report explains the cause of death of a roloway monkey (*Cercopithecus roloway*), a critically endangered primate native to the Upper Guinea forests of West Africa, that was kept for captive breeding and conservation at the Accra Zoological Garden′s primate breeding center in Ghana. *Sweetpea*, a “15‐year‐old” female Roloway monkey, was found dead on September 21, 2018, without prior signs of illness. Gross and histopathological findings revealed acute fibrinous pneumonia, septicemia, and myocardial necrosis, while bacterial culture of lung tissue and thoracic fluid yielded *β*‐hemolytic *Streptococcus* spp., implicating it as the likely cause of death. Whereas species‐level identification was not performed due to resource limitations, isolation of this species in pure culture supports its implication in this case, leaning on the published knowledge of its primary role in bacterial septicemia and pneumonia and other soft tissue infections in monkeys, even in captivity. Roloway monkeys (*Cercopithecus roloway*) are critically endangered primates native to the Upper Guinea forests of West Africa. This report describes the sudden death of a 15‐year‐old female at the Accra Zoological Garden and the subsequent diagnostic investigation, which revealed *β*‐hemolytic *Streptococcus* spp. as the causative agent of acute pneumonia and septicemia. This case underscores the need for structured health monitoring in captive breeding programs and highlights veterinary and husbandry measures crucial to conservation efforts. Additionally, a uterine leiomyosarcoma was detected, which may explain Sweetpea′s failure to reproduce despite long‐term pairing. This case highlights the importance of surveillance for beta‐hemolytic streptococci in captive monkeys, more so the endangered species.

## 1. Introduction

Roloway monkeys (*Cercopithecus roloway*) are critically endangered primates native to the Upper Guinea forests of West Africa, ranging from southeastern Côte d′Ivoire (from the River Sassandra) to southwestern Ghana [1]. Since 2008, they have consistently featured on the list of the world′s 25 most endangered primates [2, 3]. The continued decline of roloway monkeys is driven by illegal pet ownership, hunting, and habitat encroachment, all of which increase the risk of disease transmission and reduce genetic diversity in remaining populations [4–6].

Habitat destruction and encroachment further exacerbate these threats, resulting in a shrinking natural range and declining visibility of the species in formerly populated regions [7, 8]. Conservation efforts are, therefore, critical, particularly those that combine in situ and ex situ strategies.

This case report presents findings from Sweetpea′s sudden death and postmortem investigation, which revealed acute bacterial septicemia caused by *β*‐hemolytic *Streptococcus* spp. It highlights the diagnostic, management, and conservation challenges involved in maintaining critically endangered primates in captivity.

The West African Primate Conservation Action (WAPCA), a local nongovernmental organization operating in Ghana and Côte d′Ivoire, is dedicated to the conservation of endangered primates indigenous to the region [9]. In partnership with the Wildlife Division of the Forestry Commission and other stakeholders, WAPCA uses community engagement, research, education, and captive breeding programs to achieve its goals.

## 2. Case Presentation

*Sweetpea*, a 15‐year‐old female roloway monkey, was confiscated from a rural farmer in Ghana′s Western Region, where she had been kept illegally as a pet. She was enrolled in the Accra Zoological Garden on September 21, 2008, and subsequently enrolled in the Endangered Primate Breeding Center (EPBC) in Accra. EPBC is an ex situ conservation unit focused on species such as the white‐naped mangabey (*Cercocebus lunulatus*) and the critically endangered roloway monkey [10].

She was later paired with a male from the Paris Zoo under the guidance of the studbook keeper for the Diana monkey population at the Barcelona Zoo. Despite strong social bonding, reproduction did not occur.

On Friday, September 21, 2018, *Sweetpea* was found dead in her enclosure at the Accra Zoological Garden. She had no apparent clinical signs of illness and was active just hours prior to her sudden death. The carcass was double‐bagged and stored at −20°C pending necropsy and further investigation, in accordance with the zoo′s standard postmortem handling protocols.

Following the incident, her male cage mate was placed under veterinary observation for signs of illness but remained clinically healthy. Efforts to identify a suitable breeding partner are ongoing to support the continuity of the conservation program.

On October 16, 2018, the carcass was submitted to the Small Animal Teaching Hospital of the University of Ghana. A cosmetic necropsy was performed on the thawed‐out carcass by specialists in wildlife medicine and pathology, with the dual purpose of conducting a postmortem examination and preparing the carcass remains for taxidermy. Due to the cosmetic nature of the procedure, restricted sampling was performed, targeting organs with notable gross pathological changes. The necropsy procedure was consistent with standard guidelines [11, 12]. Taking full account of the case history, a thorough external examination was conducted to assess the status of hydration, body condition, surface lesions, ectoparasites, discharges from body orifices, and any evidence of trauma and other lesions. Subcutaneous tissue and associated lymph nodes were examined from the chin, entire head and neck, thorax, and the entire ventrolateral abdomen and around the limbs. Body cavities (thoracic first) were procedurally opened and examined in situ for the anatomic position of organs, abnormal effusions, or exudates. Organs were procedurally removed and systematically examined.

Nasal, oral, and tracheal swabs were collected aseptically using sterile swabs. In addition, lung tissue, fine‐needle aspirates of pleural, pericardial, and peritoneal fluids, as well as gastrointestinal contents, were obtained and submitted for bacterial culture and microbiological analysis at the Noguchi Memorial Institute for Medical Research, University of Ghana. All the samples were transported under cold chain conditions (4°C) within 2 h and subsequently processed for bacterial culture in a biosafety Level 2 laboratory, adhering to the established bacterial culture protocol. Samples were cultured on 5% sheep blood agar and incubated at 37°C under facultative anaerobic conditions with 5% CO_2_ for 24 h. Plates were examined for growth, and the morphology of colonies along with gram staining was assessed.

Tissue samples (0.5 cm thick) were taken from the lung, heart, liver, spleen, kidneys, and uterus, fixed in 10% neutral buffered formalin for histopathological evaluation. Formalin‐fixed specimens were trimmed to 2–3 mm thick, processed for histopathology in an automatic tissue processor (Leica Bio systems), sectioned at 4–5 *μ*m thickness using a semiautomated rotary microtome (Microm, HM 355S), and stained with hematoxylin and eosin (H&E) stains using Leica Autostainer XL (Leica Microsystems) connected with Leica CV 5030 for permanent cover slipping with D.P.X. mountant [13]. The slides were then carefully evaluated for lesions and other abnormalities using a light microscope (Olympus, Japan) fitted with a digital camera (AmScop 3.7 digital camera, MD500, United States). Photomicrographs of the various tissues were captured for a detailed presentation at a magnification of x400.

## 3. Results

### 3.1. Gross Necropsy Findings

The main gross findings are illustrated in (Figures 1a, 1b, 1c, 1d, 1e, and 1f). External examination revealed no overt abnormalities (Figure 1a). The subcutaneous fat was scanty gelatinous and yellowish (Figure 1b and 1c), suggestive of moderate emaciation. Approximately 20 mL of serosanguineous effusion with moderate networks of fibrin strands occurred in the thoracic cavity (Figure 1c), 5 mL in the pericardial sac, and 50 mL in the peritoneal cavity (Figure 1b). Mild to moderate, loose meshwork of fibrinous adhesions occurred on the visceral peritoneum on the surface of abdominal organs and on the parietal peritoneum on the abdominal wall.

Figure 1Gross necropsy findings in a 15‐year‐old female roloway monkey (*Cercopithecus roloway*). (a) *Sweetpea* showing unremarkable external appearance. Yellowish subcutaneous adipose tissue (yellow arrows) and accumulation of serosanguineous fluid in (b) abdominal and (c) thoracic cavities. (d) Diffusely dark red–black epicardium consistent with hemorrhagic and congested pericardium. (e) A light‐toned firm mass, suggestive of neoplasia protrudes from the wall of the uterine body (black arrow). (f) Cut surface of the uterus revealing that the mass is light colored and densely packed on cut surface and that it relates to the wall of the uterus and contributes to its thickening.

**Figure:**
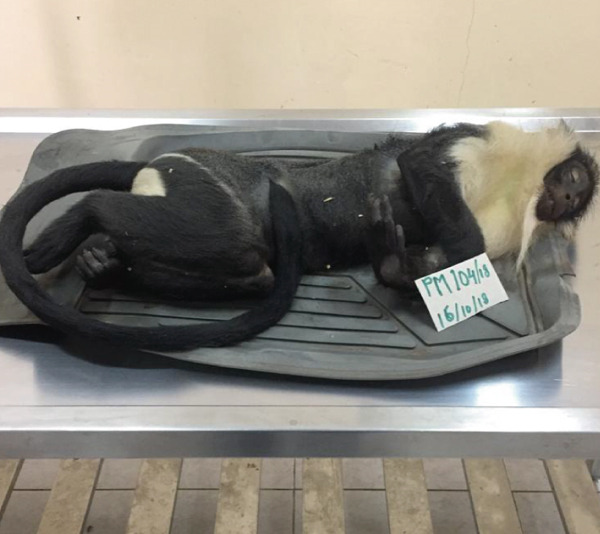
(a)

**Figure:**
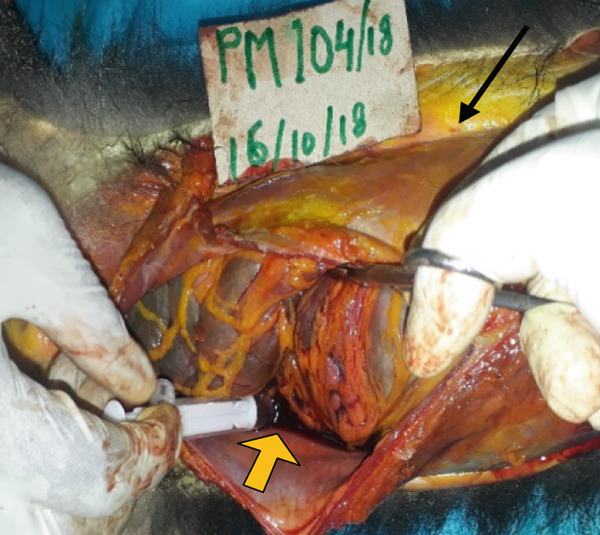
(b)

**Figure:**
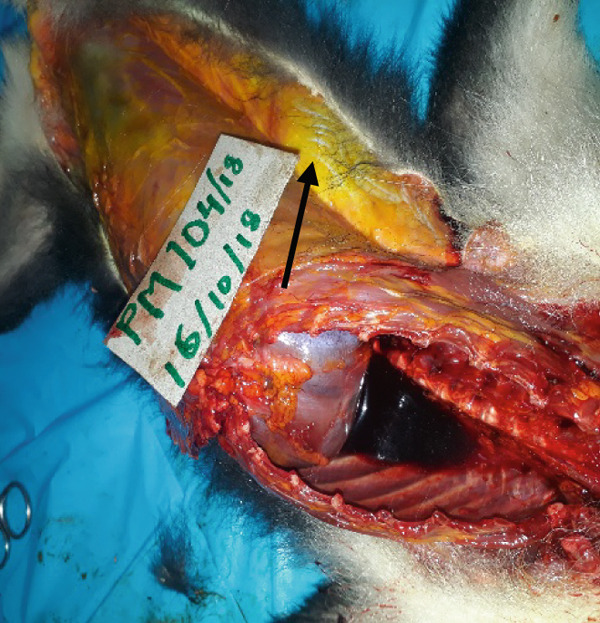
(c)

**Figure:**
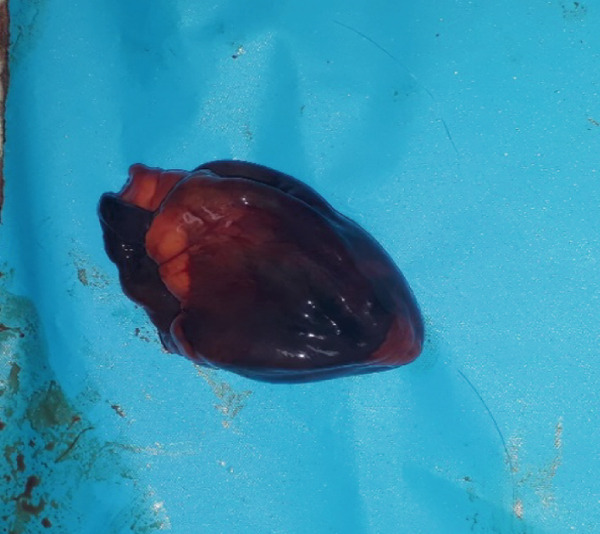
(d)

**Figure:**
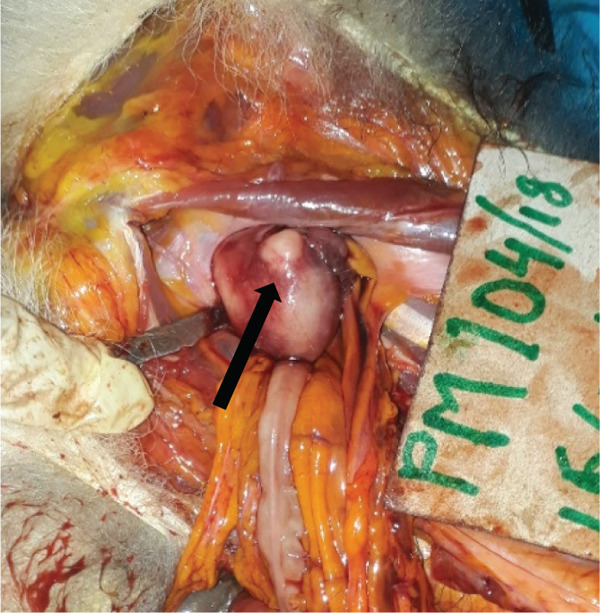
(e)

**Figure:**
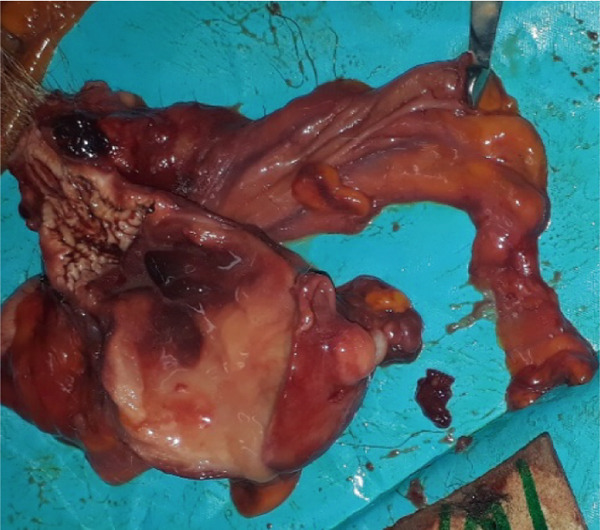
(f)

The entire lung was mildly firm in consistency with a moist foamy cut surface and had a diffuse red to black color on the pleura and into cut surfaces, all consistent with hemorrhage, congestion, and edema. Strands and webs of loosely attached fibrin occurred extensively on the pleural surface, consistent with acute fibrinous pneumonia. A loose (mild) meshwork of fibrin mildly anchored parts of the visceral pleura on the lung surface to the parietal pleura lining the thoracic wall and the heart. The heart was diffusely dark‐red on the entire epicardium, consistent with hemorrhage and congestion (Figure 1d). The liver had rounded edges, and it was diffusely dark red, which is consistent with hepatomegaly and passive congestion. Both kidneys were uniformly dark red (congested) on the capsular and into the cut surfaces. The spleen had round edges, and it was uniformly dark red on the capsular and cut surfaces consistent with splenomegaly and diffuse congestion. Dark red, tarry, viscid content oozed from its cut surface. The serosa of the stomach was dark red, consistent with congestion, and the mesenteric vessels were prominently distended. However, gastric contents appeared normal, and the contents of the entire intestines and feces in the colon and rectum were well formed.

The urinary bladder was empty and mildly but diffusely red (congested). Examination of the reproductive tract revealed no gross abnormalities in the vagina and cervix. However, the wall of the entire uterus was prominently thick and had a nodular firm mass (3 by 5 cm) that was suggestive of neoplasia on the uterine body (Figure 1e and 1f).

### 3.2. Histopathological Findings

Examination of the lungs revealed marked and extensive occurrences of homogeneous, eosinophilic substances, consistent with severe pulmonary edema and fibrin occurring in the alveoli and interstitial spaces of the lung (Figure 2a). Concurrently, there are numerous irregular empty spaces that are not delineated by an epithelium that occur in thawed‐out ice crystals in the tissues of freeze‐thawed carcasses. Multifocal atelectasis and commingling emphysema were observed, likely secondary to alveolar compression by accumulated fluid and compensatory emphysema of the intact alveoli. Multifocally, the medium‐sized arterioles in the lungs contained outstanding thrombi that were anchored to the endothelium and were extensively infiltrated by inflammatory cells, consistent with disseminating thromboembolic infection in septicemia (Figure 2b).

Figure 2(a) Lung: marked and diffuse presence of pink stained content in the alveoli and interstitial spaces consistent with fibrin and edema. Multifocal irregular empty spaces of varying sizes occur in the interstitium and correspond to ice crystals in freeze‐thaw carcasses H&E x100). (b1) Lung: the thrombus fills the entire lumen of medium‐sized artery and it is extensively infiltrated by debris of inflammatory cells and basophilic staining dust mimicking bacteria in the thrombus (H&E x100). (b2) Lung: Close‐up of 2(b1) and 2a. A thrombus fills the entire lumen of medium‐sized artery and it is extensively infiltrated by debris of inflammatory cells and basophilic staining dust mimicking bacteria in the thrombus (H&E x400).

**Figure:**
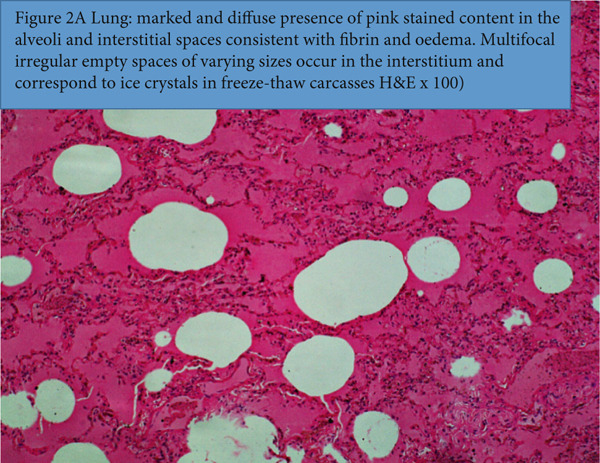
(a)

**Figure:**
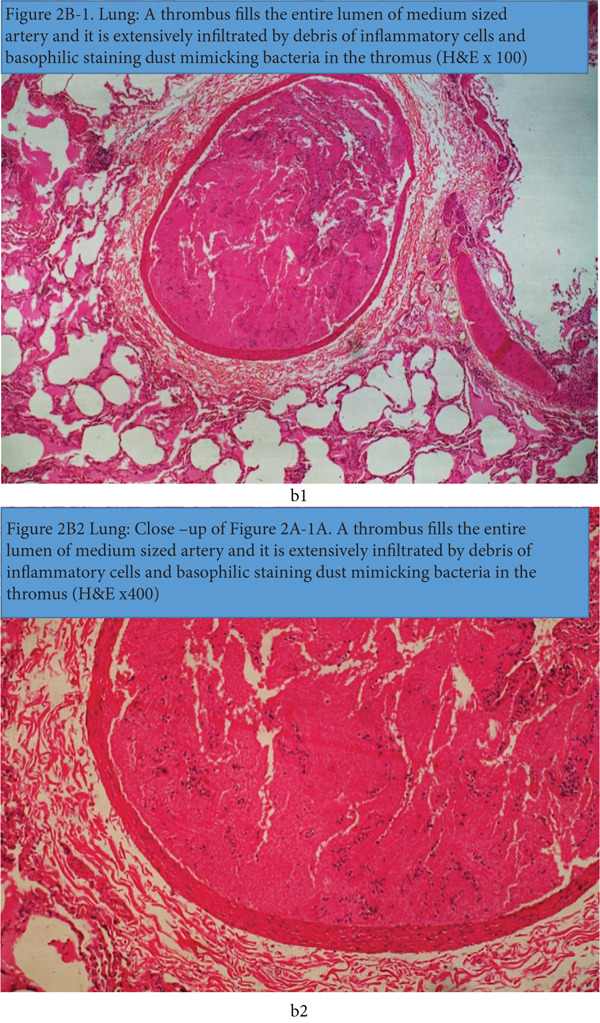
(b)

In the heart (Figure 3a, b1 and b2), there is zonal myocardial degeneration and necrosis, characterized by the presence of pink stained hyalinized coagulum of necrotic myofibres, that were clearly demarcated from the adjacent and the apparently more intact myocardium (Figure 3a). The necrotic zones also hosted debris of dead myocardial cells and the infiltrating neutrophils and mononuclear inflammatory cells (Figure 3b). Sporadically the foci of necrosis elicited and manifested focal inflammatory reaction characterized by debris of neutrophils and mononuclear cells.

Figure 3(a) Heart: multifocal to focally extensive myocardial degeneration and necrotic myocardial cells occur widely and present as eosinophilic clumps and shreds of dead myocardial cells in between more viable ones (H&E x400). (b1) Heart: a focal to focally extensive necrosis depicts debris of tissue cells and fragments of neutrophils mixed into a coagulum of fibrin (H&E x400) (arrow). (b2) Heart shows a focus of myocardial necrosis, debris of dead tissue cells infiltrated by neutrophils, and mononuclear inflammatory cells (green arrow) (H&E x100). The empty spaces within and between the intact myocytes (black arrow) are associated with thawed‐out intracellular ice crystals.

**Figure:**
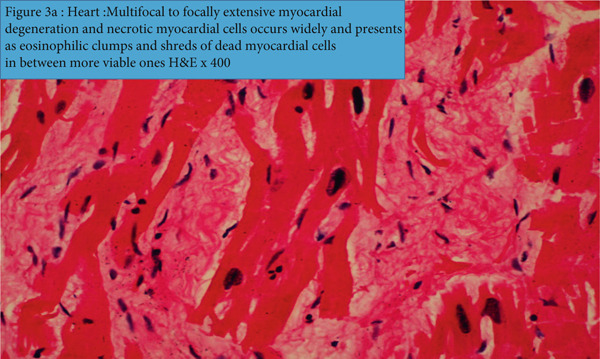
(a)

**Figure:**
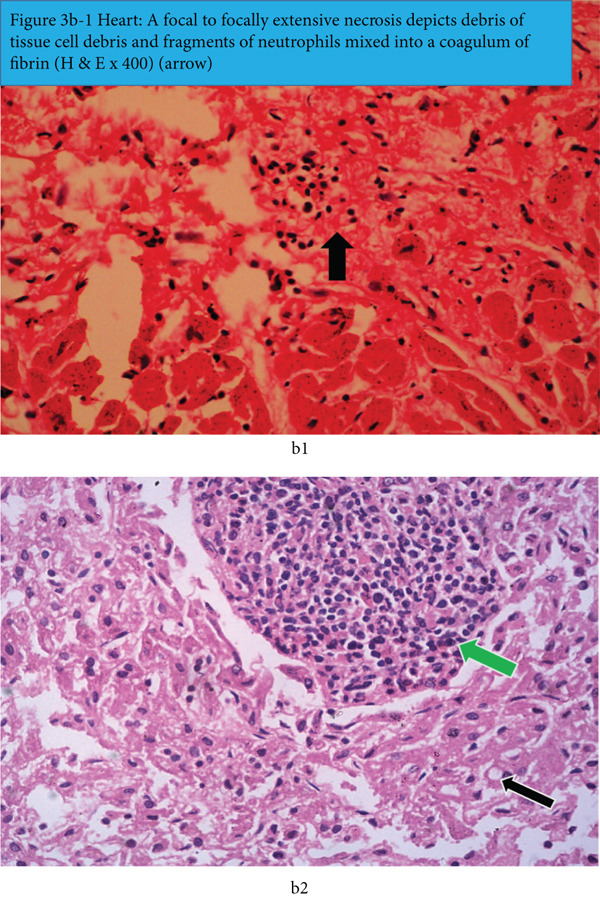
(b)

The liver (Figure 4a) displayed loss of hepatic lobular pattern, focally extensive periportal necrotic lesions, and vacuolization of the cytoplasm of more intact hepatocytes as well as the occurrence of multifocal inflammatory lesions that were characterized by infiltration with fibrin mixed with cell debris in the periportal areas and the occurrence of multifocal centers of hepatocyte necrosis (black arrow).

Figure 4(a) Liver: portal region contains fibrin and inflammatory cell debris putatively neutrophils (green arrow). A focus of necrotic tissue rimmed by fibrin is in view in the lobule to the left (black arrow, H&E x100). (b) Spleen: diffuse marked necrosis of lymphocytes has caused severe extensive depletion of the white pulp. A depleted periarteriolar lymphoid sheath is in view (arrow) (H&E x100).

**Figure:**
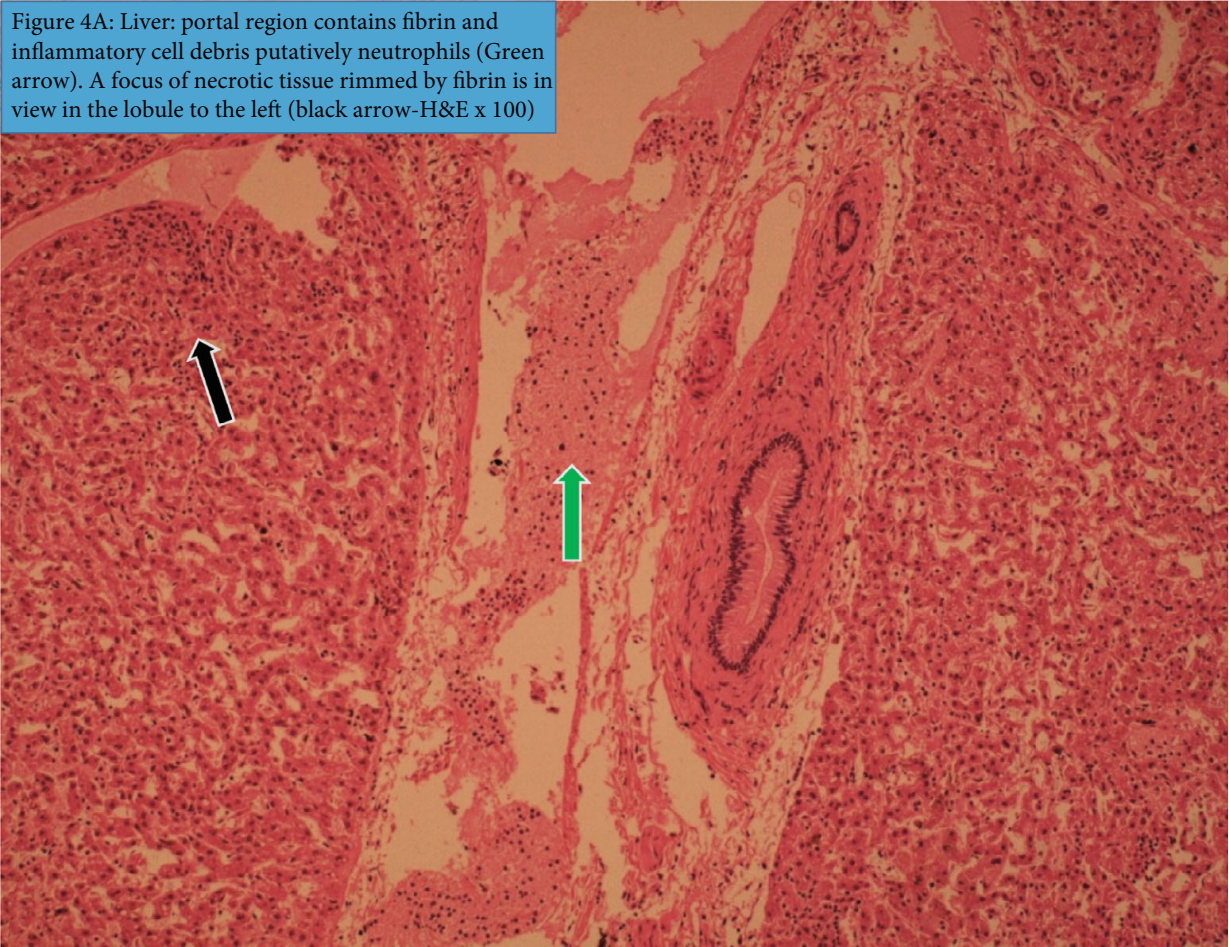
(a)

**Figure:**
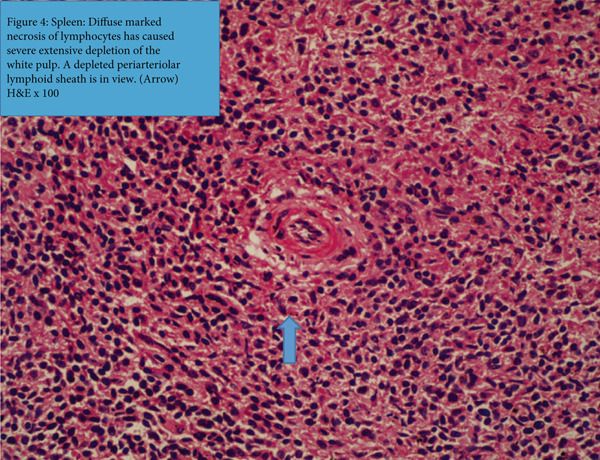
(b)

The spleen (Figure 4b), displayed widespread and marked depletion of white pulp that was caused by necrosis of lymphoid tissue, most prominently in the periarteriolar lymphoid sheaths (PALS) and marginal zones of the follicles. An increased population of macrophages occurred in the peritrabecular sinusoids, and many of the macrophages were engorged with necrotic debris in the cytoplasm.

**Figure 5:**
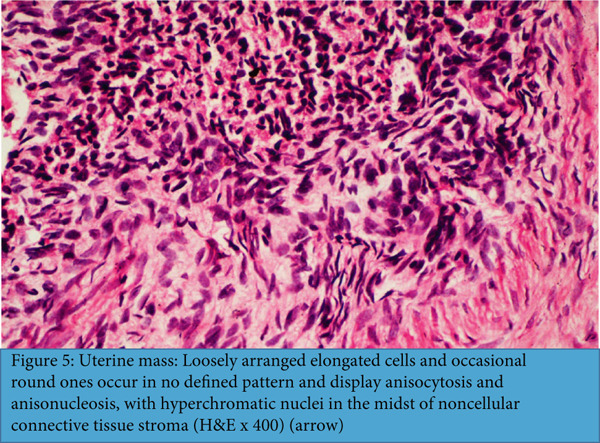
Uterine mass: loosely arranged elongated cells and occasional round ones occur in no defined pattern and display anisocytosis and anisonucleosis, with hyperchromatic nuclei in the midst of noncellular connective tissue stroma (H&E x400) (arrow).

Histological sections of the uterine mass (Figure 5) showed a neoplastic proliferation of loosely arranged, pleomorphic spindle to elongated cells, with occasional round forms. The neoplastic cells had hyperchromatic nuclei, abundant eosinophilic cytoplasm, and exhibited marked cellular and nuclear pleomorphism (*anisocytosis* and *anisonucleosis*). Occasional multinucleated cells and necrotic foci were noted. The tumor cells were not arranged in any specific pattern, neither were they compactly packed with their adjacent mesenchyme. The cells intermingled in different directions, exhibited poorly defined cell borders, and were embedded in a noncellular eosinophilic connective tissue stroma. These features are morphologically consistent with a diagnosis of uterine leiomyosarcoma.

### 3.3. Bacteriology

Clear zones were observed around colonies on the plates, indicating complete erythrocyte lysis, which suggests the presence of Beta‐hemolytic *Streptococcus* sp. isolated from the lungs and thoracic fluid. The preliminary gram staining test revealed gram‐positive cocci arranged in chains. No bacterial growth was detected in the other samples.

Species‐level identification of the isolate was not conducted due to resource limitations, and molecular characterization or antibiotic susceptibility testing was not performed. As the culture was conducted on frozen postmortem tissues, the possibility of postmortem bacterial translocation, overgrowth, or contamination cannot be entirely excluded.

## 4. Discussion

Captive breeding programs are vital to the conservation of endangered wildlife species. However, the establishment of breeding centers within tourist‐oriented facilities, such as zoological gardens, presents inherent biosecurity challenges—notably the potential for pathogen spill over between humans, domestic animals, and captive wildlife [14].

In this case, postmortem and laboratory findings identified *β*‐hemolytic *Streptococcus* spp. in the lung and thoracic fluid of a 15‐year‐old roloway monkey (*Sweetpea*), suggesting its involvement in acute pneumonia and subsequent septicemia. *Streptococcus* species are known causes of morbidity, mortality, and characteristic multisystemic, purulent, and fibrinopurulent, occasionally hemorrhagic gross and microscopic pathology, in non‐human primates [15]. Whereas the *Streptococcus* spp. in this case was not identified to the species level in the bacteriology laboratory, the sudden death, gross lesions, and histopathology findings in the lungs, heart, liver, kidneys, spleen, and other organs, along with the isolation of beta‐hemolytic *Streptococcus* spp. in the lungs and body fluids, highly incriminate this organism in the pathology of the disease that caused the death of Sweetpea. Fibrinohemorrhagic pneumonia with pleurisy and fibrinous strands in pleural and peritoneal effusions in the thoracic, peritoneal, and pericardial cavities, and the presence of microthrombi of small‐ and medium‐sized blood vessels in histopathology are consistent lesions of *Streptococcus* spp. septicemia in non‐human primates [15]. The occurrence of inflammatory cells in the pulmonary thrombus supports the argument that a coexisting hematogenously disseminating inflammatory process consistent with septicemia was a major lesion in the disease reported in this case. The thrombi were clearly delineated by an intact blood vessel wall, which suggests that autolysis had not significantly advanced in the tissue, thereby retaining cellular integrity. Furthermore, the occurrence of multifocal to focally extensive myocardial degeneration and necrosis, along with associated inflammation demonstrated in the heart, reaffirms that these were intravital reactive changes to an injury in the myocardium that could not have been caused by autolysis or freeze‐thawing. On the flip side, autolysis would present a more extensive uniform dissolution of tissue devoid of reactive inflammation and interfacing dead and viable areas as observed in the heart, liver and spleen.

Besides, the presence of focal necrotic lesions in periportal hepatocytes and fibrinous exudate with debris of neutrophils in the portal region confirms that the inflammation demonstrated in the liver was intravital and vindicates freeze‐thawing and autolysis in the development of lesions described in the liver parenchyma.

Whereas a demonstration of the presence or absence of chains of gram‐positive cocci bacteria consistent with *Streptococcus* spp. in histological sections of predilection organs (such as the lung and spleen) would have unequivocally associated the lesions in this case with the organism, it is documented from past studies that not all cases that are positive on culture correlate with the presence of gram stain demonstration of chains of *Streptococcus* species in culture [16]. From the evidence gathered in this case, the sudden death of Sweetpea without prior clinical signs and the presence of *β*‐hemolytic *Streptococcus* in lung tissue and the consistent pathology, suggest acute septicemia as the primary cause of the disease. Fibrinous exudative lesions and thrombosis described in the lungs of this case and inflammatory foci in the heart, liver, and depopulation of lymphoid tissue in the spleen described in this case are consistent with the disseminated multiple organ pathology of *Streptococcus* spp. depicted by Davis et al. in their study on the pathology of *Streptococcus* spp. in baboons [15]. Although the entire systemic pathology of the disease that caused the death of the Diana monkey reported in this case could not be performed in compliance with the owner′s request for a cosmetic autopsy, the lesions described in the lungs, liver, heart, and spleen support a differential diagnosis of streptococcal septicemia, which concurs with its pathology in humans [15] and is consistent with the use of nonhuman primate species as models for human sepsis [17]. The environmental stressors, inadequate health monitoring, and possible exposure to pathogens via fomites or contaminated food may have contributed to the onset and severity of the disease.

Histopathological examination also revealed uterine leiomyosarcoma, a malignant smooth muscle tumor. While this neoplasia was likely an incidental finding relative to the acute cause of death, it may have had significant implications for Sweetpea′s reproductive performance. Although she had been paired with a compatible male and showed signs of social bonding, no offspring were produced during her time in the breeding program. Uterine leiomyosarcomas are known to cause infertility or subfertility in domestic animals and humans due to distortion of uterine architecture, altered hormone response, or impaired implantation. It is therefore plausible that the tumor contributed to her inability to reproduce, further compounding the conservation impact of her loss.

Literature demonstrates that nonhuman primates species and humans are target hosts for *β*‐hemolytic *Streptococcus* and manifest lesions consistent with those reported in this case. At the Southwest National Primate Research Center, *β*‐hemolytic *Streptococcus* was reported as a cause of death in baboons (*Papio* spp.). Similarly, outbreaks involving *Streptococcus pneumoniae* were reported in captive chimpanzees at the Limbe Wildlife Center (LWC) and Tai National Park (TNP), where histopathological findings included purulent bronchopneumonia with diffuse infiltration of neutrophils and macrophages [14, 15]. These findings are consistent with the pulmonary pathology observed in Sweetpea.

The present case underscores the need for structured veterinary oversight, including comprehensive health monitoring, environmental hygiene protocols, food safety checks, and rigorous record‐keeping in ex situ conservation programs. These measures are crucial not only for individual animal welfare but also for the sustainability of breeding initiatives for endangered primates.

## 5. Conclusion and Recommendation

We conclude that the death of Sweetpea was due to acute pneumonia and septicemia caused by *β*‐hemolytic *Streptococcus* spp.

Additionally, the presence of a uterine leiomyosarcoma may explain Sweetpea′s lack of reproductive success during her time in the breeding program, despite being paired with a compatible mate. This underscores the need for regular reproductive and health screening in captive breeding initiatives. We also recommend strengthening partnerships with conservation NGOs, zoos, and academic institutions to support evidence‐based ex situ breeding programs

## 6. Limitations

This case report is subject to several limitations. First, no molecular or genetic sequencing was performed to further classify the isolated *β*‐hemolytic *Streptococcus* spp. to the species level. The reliance on culture from frozen postmortem tissues also raises the possibility of contamination, bacterial translocation, or overgrowth, which must be considered in interpreting the results.

Secondly, the absence of detailed veterinary health records and husbandry documentation for the affected animal significantly restricted the assessment of potential predisposing factors, including prior illnesses, environmental stressors, nutritional status, or other undiagnosed conditions. This lack of historical data limits the ability to fully reconstruct the clinical progression and contributory causes of death.

## Disclosure

All authors wrote, reviewed, edited the manuscript, and approved the submitted version.

## Conflicts of Interest

The authors declare no conflicts of interest.

## Author Contributions

Richard Suu‐Ire, Peter Gathumbi, and Samuel Asumah conceptualized the work and conducted the investigation. Peter Gathumbi was the lead veterinary pathologist. Richard Suu‐Ire, Peter Gathumbi, Mustapha Ahmed, Henry Abugri, and David Turkson conducted the investigation. Richard Suu‐Ire, Peter Gathumbi, Richard Abbiw, and Samuel Asumah wrote the original draft.

## Funding

No funding was received for this manuscript.

## Data Availability

The data that support the findings of this study are available from the corresponding author upon reasonable request.
